# Recent advances in understanding of the pathogenesis of ANCA-associated vasculitis

**DOI:** 10.12688/f1000research.14626.1

**Published:** 2018-07-19

**Authors:** Maria Prendecki, Charles D. Pusey

**Affiliations:** 1 Department of Medicine, Imperial College London, Hammersmith Campus, Du Cane Road, London, W12 0NN, UK

**Keywords:** ANCA, Vasculitis

## Abstract

Anti-neutrophil cytoplasm antibody (ANCA)-associated vasculitides (AAV) are rare systemic autoimmune diseases characterised by inflammation of small blood vessels. Recent developments have been made in our understanding of the pathogenesis of these diseases, including the pathogenic role of ANCA, neutrophils and monocytes as mediators of injury, dysregulation of the complement system, and the role of T and B cells. Current treatment strategies for AAV are based on broad immunosuppression, which may have significant side effects. Advances in understanding of the pathogenesis of disease have led to the identification of new therapeutic targets which may lead to treatment protocols with less-toxic side effects. The aim of this review is to summarise current information and recent advances in understanding of the pathogenesis of AAV.

## Introduction and background

The anti-neutrophil cytoplasm antibody (ANCA)-associated vasculitides (AAV) are a group of systemic autoimmune diseases characterised by inflammation of small blood vessels with multi-organ involvement, including the kidney, lung, nerves, gut, and ear, nose, and throat (ENT). Until 1979, it was assumed that rapidly progressive glomerulonephritis (RPGN) was caused by circulating immune complexes or anti-glomerular basement membrane (anti-GBM) antibodies. However, Stilmant *et al*. observed that many cases had no evidence of glomerular deposition of complement or immunoglobulin and were pauci-immune ^1^. Subsequently, antibody binding to neutrophil cytoplasm was shown by using serum from patients with crescentic glomerulonephritis for indirect immunofluorescence ^2^. The two main target antigens of ANCA were then identified as proteinase-3 (PR3) and myeloperoxidase (MPO), which are present in the granules of neutrophils and lysosomes of monocytes ^3– 5^. There are differing clinical syndromes associated with ANCA: granulomatosis with polyangiitis (GPA), microscopic polyangiitis (MPA), eosinophilic GPA (EGPA), and renal limited vasculitis. Around 10% of patients are ANCA negative ^6^. The aim of this review is to provide an overview of the current information and recent advances in understanding of the pathogenesis of AAV focussing on MPA and GPA rather than EGPA.

AAV is uncommon; its incidence in Europe is reported to be 13 to 20 cases per million ^7^. There is a slight male preponderance, and incidence increases with age, although peak incidence has been reported variously as 55 to 64, 65 to 74, and more than 75 years ^7– 9^. AAV is rarer in non-Caucasian or non-Asian populations, and there are differences in the incidence of different clinical phenotypes between populations. When a Japanese and UK population were directly compared, the overall incidence of AAV was similar but GPA was much less common in Japan ^10^. There is certainly a genetic basis for AAV, and this may explain some of the population differences. Two large genome-wide association studies showed an association between AAV and genetic factors, and there was a stronger genetic association with ANCA specificity than clinical syndrome, suggesting that MPO-ANCA and PR3-ANCA may be defining differing diseases. In anti-PR3 AAV, there were associations with *HLA-DP*, *PRTN3* (the gene encoding proteinase-3), and *SERPINA1* (the gene encoding a1-antitrypsin, a circulating inhibitor of PR3); anti-MPO AAV was associated mainly with *HLA-DQ* polymorphisms ^11, 12^. There are reports of an association between HLA-DRB1*15 and PR3-ANCA in African-Americans, and HLA DPB1*0401 has also been associated with PR3-ANCA disease ^13, 14^. Several studies have shown an association with a single-nucleotide polymorphism (SNP) in *PTPN22* (the gene encoding a lymphoid-specific phosphatase which is involved in T-cell activation) and GPA, although whether this SNP is also associated with MPA is less clear ^15, 16^.

There are several reported environmental associations with AAV. Infection may precede disease relapse and nasal carriage of staphylococci correlates with disease relapse in patients with anti-PR3 AAV and ENT disease ^17^. A mechanism of molecular mimicry whereby an immune response against microbial antigens cross-reacts with self tissue has been proposed ^18^. An atypical ANCA, anti-human lysosome-associated membrane protein-2 (anti-LAMP-2) antibody, was first identified in patients with pauci-immune glomerulonephritis (GN) in 1995 and has 100% sequence homology with FimH, a bacterial adhesion protein on Gram-negative bacteria. Rats immunised with FimH develop GN and antibodies which react to human and rat LAMP-2 ^18^. However, the clinical association has been reproduced in some but not other laboratories ^19, 20^. An alternative proposal involves complementary peptides of the auto-antigen. Patients with anti-PR3 AAV have been shown to have circulating antibodies to both PR3 and anti-sense complementary PR3 peptides (cPr3), suggesting that an initial immune response may be against the anti-sense peptide leading to the development of anti-idiotype antibodies which recognise PR3 ^21^.

Other environmental risk factors identified include silica, heavy metal exposure and drugs which can induce ANCA, including propylthiouracil, hydralazine, and levamisole-contaminated cocaine ^22– 24^.

## Pathogenicity of ANCA

ANCA have been shown to be pathogenic in several clinical and pre-clinical studies. There is a reported case of maternal–foetal transfer of anti-MPO ANCA resulting in neonatal renal disease and pulmonary haemorrhage shortly after birth ^25^. Levels of ANCA have been shown to correlate with disease activity in some but not all case series with better correlation in patients with renal disease ^26^. Removal of antibodies with plasma exchange has been shown to improve prognosis in severe AAV ^27^, and depletion of B cells with rituximab has been shown to be effective in induction and maintenance of remission ^28– 30^.

Some of the best evidence for the pathogenesis of ANCA comes from a passive transfer model of anti-MPO AAV. MPO-deficient mice are immunised with mouse MPO and develop high-titre anti-MPO antibodies. Transfer of these antibodies into wild-type or Rag2 mice (which lack lymphocytes) results in the mice developing severe vasculitis with crescentic GN and pulmonary haemorrhage, demonstrating that MPO-ANCA alone are sufficient to induce disease ^31^. Neutrophils were shown to have an essential role in disease pathogenesis in this model; depletion of neutrophils prior to transfer of antibodies prevented the development of disease ^32^. ANCA have also been shown to be pathogenic in an autoimmune rat model of AAV, experimental autoimmune vasculitis in the susceptible WKY rat strain. Rats are immunised with human MPO in complete Freund’s adjuvant and also receive two doses of intraperitoneal pertussis toxin as an immune adjuvant. Animals develop polyclonal anti-MPO antibodies with pauci-immune vasculitis and pulmonary haemorrhage. Intra-vital imaging in this model showed increased leucocyte adhesion and transmigration at the endothelium in response to CXCL1, and this could also be observed in healthy animals following infusion of anti-MPO IgG isolated from rats with disease, supporting a role for the pathogenicity of ANCA ^33^. The pathogenic role of PR3-ANCA is less well defined and this is owing, at least in part, to the difficulty in developing animal models of anti-PR3 AAV. An attempt to create a passive transfer model analogous to the one using anti-MPO antibodies resulted in no features of vasculitis and only a mild inflammatory response to tumour necrosis factor (TNF) in the skin ^34^. This is potentially due to a lack of PR3 expression on the surface of unstimulated mouse neutrophils and a lesser degree of sequence homology between mouse and human PR3 than there is for MPO ^35^.

Despite evidence for the pathogenicity of ANCA, the relationship between ANCA and active vasculitis is complex and ANCA are not always pathogenic. ANCA can persist in remission, can recur without evidence of clinical relapse, and have been identified in healthy individuals. Natural anti-MPO antibodies are of lower avidity and titre than are antibodies from patients with AAV ^36^. The IgG subclass of ANCA may also be important. *In vitro*, IgG3-ANCA has been shown to be more effective than other IgG subclasses at activating neutrophils, although in other clinical studies the IgG subclass of ANCA did not correlate with disease severity ^37, 38^. Epitope mapping to identify the pathogenic epitopes of both PR3 and MPO have been carried out. One study using epitope excision and mass spectrometry identified a linear epitope on MPO at residue 447–459 that was limited to patients with disease; interestingly, when the three-dimensional structure of MPO was visualised, this epitope was close to epitopes seen in individuals with natural antibodies, leading the authors to suggest that pathogenic ANCA arise by a process of epitope spreading. In this study, IgG purified from patients with ANCA-negative vasculitis was able to bind to an MPO epitope, and it was suggested that competition for binding in immunoassays by a fragment of caeruloplasmin may be why ANCA cannot be detected in these patients ^39^.

## ANCA-induced activation of neutrophils and monocytes

The ability of ANCA to bind to and activate neutrophils causing degranulation and production of reactive oxygen species (ROS) was first shown nearly 30 years ago ^40^. Since then, several *in vitro* studies have shown that neutrophils which have been primed with TNFα, lipopolysaccharide (LPS), or complement (C5a) undergo activation and degranulation and mediate endothelial cell damage when stimulated with MPO or PR3-ANCA ^41, 42^. ANCA binding to neutrophils has also been shown to activate intracellular signalling pathways leading to altered adhesion molecule expression and conformational changes which promote neutrophil adhesion and transmigration at the vascular endothelium ^43^. Both the ANCA antigen-binding site and binding to Fcγ receptors on the surface of primed neutrophils and monocytes have been identified as mechanisms by which ANCA activates these cells.

ANCA have also been shown to be mediators of NETosis, a form of neutrophil cell death with release of neutrophil extracellular traps (NETs). NETs have a DNA backbone with a variety of pro-inflammatory proteins, including histones, high-mobility group box 1 (HMGB-1), neutrophil elastase, calprotectin, MPO, and PR3 ^44^. NETs have been shown to be present at sites of tissue damage in AAV, and patients also have increased levels of NETs in the circulation ^45^. NETs may play a pathogenic role in AAV; they can cause activation of dendritic cells and autoreactive B cells, endothelial damage, and complement activation ^46, 47^. NETs may also play a role in the loss of tolerance to ANCA antigens; one study has shown that dendritic cells activated by NETs induce loss of tolerance to both MPO and PR3 ^48^. Neutrophils from patients with AAV undergo more spontaneous NETosis than those from healthy controls, but ANCA can also induce this process. The exact mechanism by which ANCA induce NETosis is unclear but is thought to require binding of both Fcγ receptors and the ANCA target antigen on the cell surface ^49^.

Although many studies have focussed on neutrophils and their interactions with ANCA in the pathogenesis of disease, monocytes may also play a role in mediating AAV. Monocytes express ANCA antigens, and stimulation of monocytes *in vitro* with ANCA leads to cytokine production and generation of ROS ^50, 51^. Monocytes from patients with AAV have been shown to express higher levels of CD14, the LPS receptor, than monocytes from patients in remission or healthy controls, suggesting an increased cell activation state in patients with AAV ^52^. Circulating monocytes from patients with active AAV have also been shown to express higher levels of cell surface markers which are essential for interaction between leucocytes and the endothelium ^53^. Recent studies have shown that monocytes and macrophages are the predominant cells in glomeruli in renal biopsies from patients with AAV ^54, 55^. In one study using the passive transfer model of mouse anti-MPO AAV, depleting monocytes decreased glomerular crescent formation but had no effect on urinary abnormalities ^56^.

## Complement and AAV

There is increasing evidence for a role for complement in the pathogenesis of AAV. In the antibody transfer model of mouse anti-MPO AAV, mice deficient in C5 or those depleted of complement by pre-treatment with cobra venom did not develop disease. C4-deficient mice were not protected, suggesting a role for the alternative rather than the classic pathway ^57^. Mice deficient in C5aR are protected from disease, and mice with knock-in of the human C5a receptor treated with an antagonist of human C5aR (CCX168; avacopan) showed decreased disease severity ^58, 59^. There is also evidence for a role for complement from *in vitro* studies; C5a can prime neutrophils to respond to stimulation by ANCA, and this may be due to its actions at the C5aR ^42^. The interaction of C5a with its other receptor, C5L2, is more complex, and some studies report that it has a pro-inflammatory role *in vitro* but knockout of C5L2 resulted in more-severe disease in mouse anti-MPO AAV ^59, 60^. It has also been shown that both ANCA-stimulated neutrophils and NETs can activate the alternative pathway of the complement system, leading to a positive feedback loop ^47, 57^. There is evidence of complement deposition, such as C3d and factor B, at sites of tissue inflammation in patients with AAV and kidney deposition of Bb (a marker of activation of the alternative pathway) correlated with pathological severity of disease ^61^. Plasma levels of C3a, C5a, soluble C5b-9, and Bb were higher in patients with active AAV than in those in remission ^62, 63^. In one study, patients with lower circulating C3 levels were shown to have poorer outcomes in terms of both patient and renal survival ^64^. Blockade of C5 cleavage with eculizumab has been reported as treatment for AAV in one case report. It was used as add-on therapy to cyclophosphamide with good renal recovery, although unfortunately the patient developed non-Hodgkin lymphoma, thought to be unrelated to the eculizumab, and died from sepsis following chemotherapy ^65^. A recently published phase II trial has shown that avacopan was effective in replacing high-dose glucocorticoids for induction of remission when added to cyclophosphamide or rituximab ^66^. A phase III trial of this treatment approach is currently recruiting (ADVOCATE, ClinicalTrials.gov Identifier: NCT02994927).

## B cells and AAV

B cells have a central role in AAV in that they produce ANCA, and levels of activated B cells have been shown to correlate with disease activity ^67^. Depletion of B cells with rituximab has been shown to be effective in inducing and maintaining disease remission ^28, 29^. The return of B cells after rituximab may predict relapse of AAV, and it has been shown that following induction of remission with rituximab and cyclophosphamide, the return of B cells has a high negative predictive value for relapse but a poor positive predictive value ^68, 69^. It may be that the phenotype of the repopulating B cells is important in predicting relapse, and one study suggested that those who repopulate with a low percentage of CD5 ^+^ B cells have a shorter time to relapse ^70^. Several studies have shown differences in B-cell subsets between patients with AAV and healthy controls. One study reported a memory B-cell subset with higher CD19 expression in patients with AAV, suggesting that these may represent autoreactive B cells ^71^. Regulatory B (B _reg_) cells skew T-cell differentiation towards regulatory T (T _reg_) cells and away from T helper 1 (T _H1_) and T _H17_ phenotypes and decrease B cells which are producing ANCA ^72^. Several studies have shown decreased B _reg_ cells in patients with AAV as defined by cell surface markers such as CD5, CD24, and CD38 ^73, 74^. *In vitro*, neutrophils stimulated with ANCA release B-cell survival factors such as B lymphocyte stimulator (BLyS) and a proliferation-inducing ligand (APRIL). In one study, incubating B cells with supernatant from ANCA-stimulated neutrophils or with recombinant BLyS resulted in increased B-cell survival ^75^. Several studies have reported higher levels of BLyS in patients with AAV, and some have shown that levels correlated with disease activity and ANCA titre and decreased following treatment ^76, 77^. Following rituximab treatment, serum BLyS levels have been shown to increase both in patients with AAV and in patients with other auto-immune diseases ^75, 78^. One study has shown that a SNP in BLyS predicted which patients were more likely to relapse following rituximab and had earlier return of B cells after treatment. The authors suggest that this SNP may result in higher baseline BLyS or increase in BLyS after B-cell depletion ^79^. This may suggest a potential role for targeting BLyS as maintenance treatment of AAV following induction treatment with rituximab. A phase III trial which added anti-BLyS treatment with belimumab to azathioprine and steroids for maintenance of remission did not show any reduction in risk of relapse; however, in the subgroup of patients who received rituximab as an induction agent, belimumab did reduce relapse rate, although this was not significant ^80^.

## T-cell immunity and AAV

T cells are present in glomeruli and the tubulointerstitium in renal biopsy tissue from patients with AAV, suggesting that T-cell responses are pathogenic. Studies have shown that patients with AAV have defective T _reg_ cell suppressive function ^81^; one study has also shown increased frequency of a CD4 ^+^ T-cell subset that is resistant to the suppressor effects of T _reg_ cells ^82^. In a small group of patients, anti-thymocyte globulin was used as a successful treatment for refractory GPA ^83^. Additionally, differential T _H_ cell polarisation has been described in AAV, such that patients with active and systemic disease are more likely to have a T _H2_ response ^84^.

In the mouse passive transfer model of anti-MPO AAV, CD4 ^+^ T cells have been used to transfer disease, demonstrating a role for T cells in pathogenesis. Mice pre-immunised with CD4 ^+^ T cells from MPO-immunised, B-cell-deficient, MPO-deficient mice developed greater severity of GN after induction of disease with MPO-ANCA compared with mice immunised with OVA-sensitised CD4 ^+^ cells ^85^. In a model of anti-MPO AAV in which mice are immunised with MPO followed by a subnephritogenic dose of anti-GBM globulin, depletion of CD4 ^+^ cells decreased disease severity with no effect on ANCA titres ^86^. This model of disease has been used to identify pathogenic epitopes for both CD4 ^+^ and CD8 ^+^ T cells, and these epitopes have been used to induce disease ^87, 88^.

The T _H17_ axis may also be involved in the development of ANCA; serum interleukin-23 (IL-23) and IL-17 are raised in the serum of patients with acute AAV, and in one study IL-23 levels correlated with disease activity ^89^. IL-23 induces T-cell differentiation into the T _H17_ subset and enhances the production of IL-17 from these cells. Stimulation of neutrophils by ANCA has been shown to induce the production of IL-17 ^90^, and in one study IL-17-deficient mice were protected from MPO-ANCA-induced disease ^91^.

## Granuloma formation

Granulomatous disease is frequently seen in isolated and systemic GPA. Early granuloma formation is typified by activated neutrophils forming micro-abscesses and only scattered multinucleated giant cells. Later granulomas consist of a central necrotic area with multinucleated giant cells at the margin and surrounding dendritic cells, T lymphocytes, B lymphocytes, and plasma cells forming a follicular structure of ectopic lymphoid tissue ^92, 93^. The mechanisms initiating granuloma formation have not been fully identified, but there is some evidence that granulomatous inflammation is being driven by T cells producing T _H1_ cytokines ^94^. It has also been shown that APRIL and BLyS are present in granulomas along with activated B cells, leading some authors to suggest that close association of B cells with PR3-positive cells within granulomas could lead to initiation or maintenance of anti-PR3 responses ^95^. In an *in vivo* model of xenografted nasal mucosa from patients with GPA to mice, tissue damage was shown to be mediated by fibroblasts ^96^.

## Conclusions

The pathogenesis of AAV is complex and remains incompletely understood. Recent advances have been made in our understanding of the mechanisms of both the development of auto-immunity and inflammation leading to tissue damage. Our understanding of the generation of the auto-immune response is incomplete but may well involve molecular mimicry and dysregulation of both B and T cells. There is substantial evidence for the pathogenicity of ANCA, and neutrophils are both the target of ANCA and mediators of endothelial injury. NETs in particular have been shown to mediate tissue damage but also could be involved in the loss of tolerance to ANCA. Advances in understanding the role of the alternative pathway of the complement system in AAV have led to clinical trials of novel therapeutic agents. Further understanding of the mechanisms of disease may lead to the use of other novel therapeutics such as molecules to block NETosis, BLyS inhibitors, or monoclonal antibodies against IL-17 or IL-23.
